# GC-FID and HPLC-DAD Methods for the Determination of Menadione Sodium Bisulphite Directly and by Converting Menadione Sodium Bisulphite to Menadione in Pharmaceutical Preparation 

**Published:** 2014

**Authors:** Fatma Demirkaya-Miloglu, Yucel Kadioglu, Onur Senol

**Affiliations:** *Department of Analytical Chemistry, Faculty of Pharmacy, Ataturk University, 25240, Erzurum, Turkey.*

**Keywords:** Menadione, HPLC-DAD, GC-FID, Pharmaceutical

## Abstract

was performed in both direct analysis of MSB and analysis of MN by converting MSB to MN with sodium carbonate. GC-FID method was carried out on the HP-5 capillary column GC-FID and HPLC-DAD methods were developed for determination of menadione (MN) and menadione sodium bisulphite (MSB). By means of each method, quantitative analysis of MSB in commercial pharmaceutical using nitrogen gas. HPLC-DAD method was achieved on the reversed phase C_8_ column by using a mobile phase consisting methanol and water. The calibration curves of GC-FID and HPLC-DAD for both analytes were linear in the same concentration range (0.5–20 μg/mL). Both methods were validated in terms of precision, accuracy, recovery and limits of detection (LOD) and quantitation (LOQ). Although LOD values of HPLC-DAD method (0.010 μg/mL for MN and 0.005 μg/mL for MSB) is lower than obtained values with GC-FID method (0.04 μg/mL for MN and 0.06 μg/mL for MSB), both methods gave similar and favorable results in terms of precision and accuracy. The Student's t-test was applied to investigate the significant of the different between the results of MSB determination with direct analysis of MSB and analysis of MN by converting MSB to MN by means of GC-FID and HPLC-DAD method in dosage form.

## Introduction

**Figure 1:**
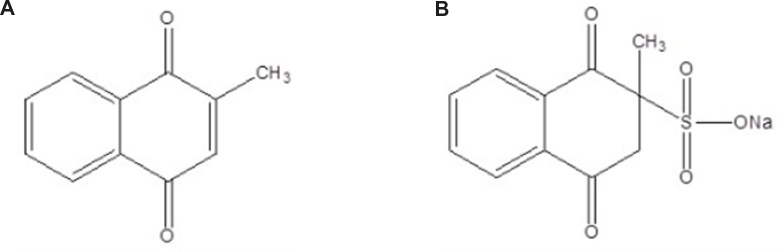
Chemical structure of (A) MN (B) MSB

Menadione (2-methyl-1,4-naphthoquinone, MN) (Figure 1A) or vitamin K_3_ is a fat soluble vitamins. Menadione sodium bisulphite (MSB) (Figure 1B) is also a synthetic analogue of vitamin K that acts as a provitamin. This compound is water-soluble salt of MN and it is used extensively as synthetic vitamin K_3 _supplements in food and pharmaceutical (1,2). MN and MSB, like other compounds in vitamin K series, are used as a required cofactor in the synthesis of blood clotting and in bone metabolism (1,3). In addition, the antitumor action of vitamin K_3_ has been under investigation. Recent investigations indicate that MN exhibits antitumor activity against both malignant cell lines and a variety of human tumor cells at relative high dose (4,5). The mechanism of MN against cancer can be explained by occurring oxidative stress in redox-cycling of the quinone to produce reactive oxygen species (ROS) such as the hydroxyl radical, superoxide radical and hydrogen peroxide. The increased redox-cycling of MN and the production ROS suppress the oxidative capacity of the cell, resulting in cell death (1,6).

Different analytical techniques including spectrophotometry (7-9), spectrofluorimetry (10-12), voltammetry (13), chemiluminescence (14,15), potentiometry (16), liquid chromatography (17-19) and gas chromatography (20) for MN or MSB determination have been described in the literature. These methods have been applied to the determination of MN or MSB in pharmaceutical preparations. Both MN and MSB have no fluorescence property. Therefore, MN and MSB must be reacted with different chemical reagents in order to give fluorescence property for the spectrofluorimetric methods. Both MN and MSB had to be derivatized in order to form a colored complex which led to complicated operations and time consuming for spectrophotometric methods too. The liquid chromatography methods with different detections such as ultraviolet (17), fluorescence (18) and electrochemical (19) have been widely employed. The columns packed with powdered zinc due to lack fluorescence properties of MN or post column derivatization were used in fluorimetric determination.

The reduction prior to a fluorimetric detection has been achieved by an electrochemical (21) or a photochemical (18) reduction. In this case, the complex and difficult systems and extra reagent consumption were required. The electrochemical detection was also used due to the highly reducible property. However, this detection was not preferred so that MSB might cause strong background current. In addition, baseline drift did not cause a problem in determination of MSB in the electrochemical detection with series dual electrodes developed by Liu *et al. *(19). C_18_ column was used in all reversed phase liquid chromatography methods in literature. Besides, normal phase liquid chromatography method was found for determination of MSB in animal feed (17). A gas liquid chromatographic method has been reported (20) but we were unable to reach detailed information about this. Terminally, the cerimetric titration method for determination of MN was described by the British Pharmacopoeia (22).

This paper describes a simple GC-FID and HPLC-DAD method without extra equipment and reagent for analysis both direct analysis of MSB and analysis of MN by converting MSB to MN in pharmaceutical preparation. 

## Experimental

*Chemicals and reagents*

MN and MSB used as reference materials, diazepam and menatetrenone as internal standard (IS) and anhydric sodium carbonate were purchased from Sigma Company (USA). The high purity acetonitrile, hexane, methanol and concentrated HCl were obtained from Merck Company (Germany). Milli-Q reagent water (Millipore, USA) was used. Libavit K^®^ ampoule containing about 20 mg MSB/2 mL (*i.e*. 12.2 MN/2 mL) was obtained from the local market.

*Instrumental parameters*

*For GC-FID method: *An Agilent 6890N Network gas chromatograph was equipped with a split/splitless injector, flame-ionization detector (FID) and an Agilent 7683 series auto sampler. The HP-5 capillary column with 0.25 µm film thickness coated with 5% phenyl, 95% dimethylpolysiloxane phase (30 m x 0.320 mm I.D., USA) was used for the analysis. The carrier gas was nitrogen adjusted to deliver a column flow-rate of 2 mL/min at the initial oven temperature. The hydrogen and synthetic air flow rates were set at 40 and 400 mL/min, respectively, for the detector with a make-up gas (nitrogen) flow-rate of 40 mL/min. The injection inlet and detector temperature were maintained at 300 °C. For both MN and MSB analyses, the column temperature was held at 150 °C (initial temperature) for 1 min and from 100 to 260 °C at a rate of 30 °C/min and thereafter to 300 °C at a rate of 50 °C/min with a 3 min final holding time.

*For HPLC-DAD method:* The analyses of MN and MSB were achieved by a Thermoquest Spectra System P 1500 coupled with an Agilent Extend C8 analytical column (150 mm × 4.6 mm I.D., 5 μm) maintained at 30 °C. The isocratic elution with a mobile phase of methanol and water (60:40, v/v) was used for MSB analysis. The gradient elution with a mobile phase including methanol and water was used for MN analysis. A gradient elution was carried out as follows: 90 % methanol was used in the first four minute and then methanol percentage was linearly increased to 100 % during 0.5 min, then to 100 % during next 7.5 min. The flow rate was 1 mL/min. The MN and MSB were monitored at the UV wavelength of 330 nm and 230 nm, respectively.

*Preparations of the standard solutions*

1) MN standard working solutions (0.5-20 QC) solutions (prepared by diluting stock solution (μ with hexane for both chromatographic methods.

2) Stock solution of MSB was prepared at 2 mg/mL concentration in acetonitrile for GC-FID method and deionized water for HPLC-DAD method and then prepared standard working solutions (the range of 0.5-20 and QC samples (diluted with their solvents. 

3) 2.5 g/mL of diazepam solutions were prepared in acetonitrile which were used as IS for proposed chromatographic methods. 

4) Two different concentrations of menatetrenone which were 22.5 g/mL (for GC-FID method) and 5 g/mL (for HPLC-DAD method) were prepared in hexane for proposed chromatographic methods. Both of them were used as IS for proposed chromatographic methods.

5) A 0.01 M hydrochloric acid solution was made by dilution of concentrated hydrochloric acid.

6) An anhydric sodium carbonate solution was prepared at % 10.6, w/v concentration in deionized water.

7) Libavit K^®^ (Mefar Drug Company, Turkey; in solution, per 1 mL: 10 mg MSB and excipients: potassium meta bisulphate (6 mg), sodium chloride (12.6 mg) and injection water (2 mL)).

*Procedure* *of converting of MSB to MN*

MSB is converted to MN at pH>11 (10 mg MSB is equivalent 6.1 mg MN) (14). In order to convert MSB to MN, procedure mentioned below should be followed: firstly, standard working solutions of MSB and menatetrenone were added to 0.2 mL 0.01 M HCl solution and vortexed for 10 min and then 0.5 mL anhydric sodium carbonate solution (10.6 %) (to shift the MSB to the water insoluble MN) and 2 mL n-hexane (to extract MN and IS from the water phase) were added in a centrifuge tube (A). Secondly, working standard solutions of MN and menatetrenone were added to 0.2 mL 0.01 M HCl solution and vortexed for 10 min and then 0.5 mL anhydric sodium carbonate solution (10.6 %) (to provide the same condition) and 2 mL n-hexane (to extract MN and IS from the water phase) were added in a centrifuge tube (B). A and B solutions were vortexed for 5 min and centrifuged at 3000 g for 30 min. Finally, upper n-hexane layers were filtered through phonomenex 0.45 µm pore size (25 mm filter) and transferred to an autosampler vial for analysis. 2 µL and 20 µL volume was injected into the GC-FID and HPLC-DAD systems, respectively.

*Preparations of the drug* *solutions*

*For direct analysis of MSB:* 2.0 mL of Libavit K^®^ containing 20.0 mg MSB was transferred to a 100 mL volumetric flask. 50 mL suitable solvent was added and the flask was sonicated. The flask was filled with acetonitrile and the final concentration was 200 µg/mL. The stock solution was diluted with acetonitrile in concentration of 25 and 50 ug/mL. 1 mL of obtained solutions was transferred to a 10 mL volumetric flask and 0.25 mL diazepam (100 µg/mL) was added and the flask was filled with acetonitrile. The drug mixtures containing 2.5 and 5 µg/mL MSB and 2.5 µg/mL diazepam were analyzed by using GC-FID and HPLC-DAD procedures.

*For analysis of MN by converting MSB to MN:* 1.0 mL of Libavit K^®^ containing 10.0 mg of MSB (i.e 6.1 mg MN) was transferred to a 100 mL volumetric flask. 50 mL 0.01 M HCl solution was added and the flask was sonicated. The flask was filled with 0.01 M HCl solution and the final concentration of MSB was 100 µg/mL. Two different concentrations of MSB which were equal to a 6.1 µg/mL and 12.2 µg/mL of MN were prepared from this stock solution. After that menatetrenone and these drug solutions were transferred into centrifuge tubes and then 0.5 mL anhydric sodium carbonate solution (10.6 %, w/v) and 2 mL n-hexane were added. This solution was vortexed for 5 min and centrifuged at 3000 g for 30 min. The upper n-hexane layers were filtered and then the drug mixture containing 6.1 µg/mL and 12.2 µg/mL of MN and 22.5 µg/mL (for GC-FID method) and 5 µg/mL (for HPLC-DAD method) of menatetrenone were analyzed by using proposed procedures.

*Method validation*

The specificities of chromatographic methods were determined by accurately measuring the analyte response in the presence of IS and all potential sample components. The linearity was shown by plotting the peak-area ratio of analyte values (MN or MSB) to their IS values) versus concentration of analyte. The intra-day precision was determined by analyzing six replicates of QC samples in single day. The inter-day precision was determined by analyzing the QC samples on three separate days. The intra-day and inter-day precision were defined as the percent relative standard deviation (RSD %) and the accuracy was defined by calculating the relative error (RE). For the recovery study, aliquots of a synthetic ampoule solution were spiked separately with known quantities (QC samples) of standard MSB or MN and then analyzed by the proposed methods. All of measurements were repeated six times. The recovery data were determined by comparing the observed peak-area ratio to those of non-processed standard solutions. The sensitivities of methods were point out the limit of detection (LOD) and limit of quantification (LOQ). LOD was defined as the lowest concentration of the drug resulting in a signal-to-noise ratio of 3:1. In order to calculate LOQ, signal-to-noise ratio of 10:1 formula was used. The stabilities of MN and MSB were evaluated with HPLC-DAD method by analyzing QC samples at concentrations including the low, medium and higher ranges of calibration curve for different temperatures [room (25 °C), refrigerator (4 °C) and frozen (-20 °C)] and times (6 h, 24 h, 48 h and 60 h). The results were evaluated by comparing these measurements against standards and expressed as percentage deviation and it was accepted as mean recovery of analytes ±10 values were stable.

*Statistical analysis of the results obtained from proposed methods*

All statistical calculations were performed with the Statistical Product and Service Solutions (SPSS) for windows, version 11.5. If calculated p-values are 0.05 or less, correlations were considered statistically significant. 

## Results and Discussion

*Optimization of GC-FID conditions*

Both MN and MSB were analyzed with the same GC-FID method. A HP5 capillary column was selected for analysis of both MN and MSB. To achieve the requested sensitivity, a split less inlet was used for sample injection. Split less inlet purge delay time was set at 1 min, by which, the majority of the injected sample was introduced into the column and the reproducibility of peak heights for both analytes and their IS was maximized. Detector temperatures ranging from 300 to 350 °C had no effect on the peak heights and areas of MN and MSB. Therefore, a temperature for FID detector was selected as 300 °C. The peak heights and peak areas of analytes and IS increased when the injection inlet temperature raising from 200 to 300 °C, after this temperature, there was no increase observed in peak heights and peak areas of the samples. There was no evidence to show thermal decomposition of analytes and their IS with the inlet temperature up to 300 °C. Because of that, an inlet temperature of 300 °C was selected to achieve a better assay sensitivity and reproducibility. Under these chromatographic conditions, the system precision determined by injecting prepared sample five times was found to be no greater than 0.5 % in most cases.

*Optimization of HPLC-DAD conditions*

In order to investigate a more convenient and simple mobile phase, several solvent mixtures containing acetonitrile or methanol and water were examined. Run time of both MSB with diazepam and MN with menatetrone increased with addition of acetonitrile into the mobile phase. So, methanol was employed as an organic modifier. While MN and diazepam was analyzed with isocratic elution, MSB and menatetrenone was analyzed with gradient elution. In both cases, water and methanol in different ratios were used for elution. In addition to this, Different flow rates (0.5, 0.75, 1 and 1.5 mL/min) and column (ambient, 20 ºC, 30 ºC, 35 ºC) temperatures were tested. It was found that both analytes excellently eluted at a flow-rate of 1 mL/min and column temperature of 30 ºC, appropriately.

*Optimization of pH while converting MSB to MN*

**Figure 2:**
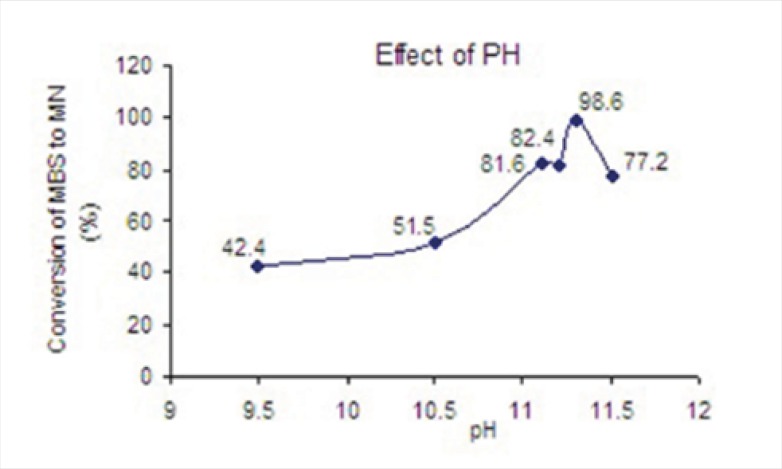
The effect of pH on conversion of MSB to MN

The effect of pH on conversion of MSB to the water insoluble MN was examined at various pH values at 9.50, 10.50, 11.10, 11.20, 11.30 and 11.50. As it was seen in Figure 2, the best condition for conversion was obtained at pH= 11.30. 

*Validation of GC-FID method* *applied for analysis of MSB and MN*

The developed GC-FID method appears to be specific for both direct analysis of MSB and analysis of its converted form (MN) in drug products. While the retention times determined for the peak of MSB and diazepam were 4.7 min and 7.8 min, respectively, these times for the peak of MN and menatetrenone were 4.6 min and 12.7 min. The total run times of the analysis were 8 min and 13 min, respectively. 

**Figure 3:**
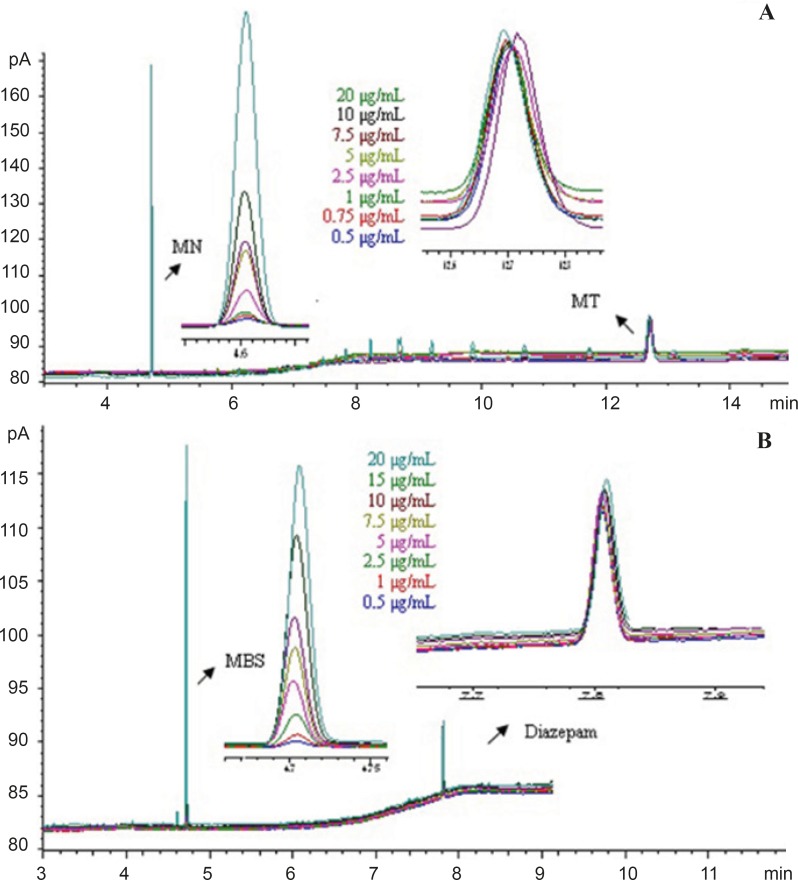
GC-FID chromatograms of obtained concentration in calibration graph of (A) MN standard solution of containing menatetrenone (B) MSB standard solution of containing diazepam.

Figure 3A and 3B show the overlay of typical chromatogram obtained from the GC-FID analysis of MSB and MN in the concentration of 0.5-20 μg/mL containing IS. 

The linear calibration range was in the concentration range of 0.5–20 μg/mL for both analytes. The coefficient of correlation (R) and regression equations for MSB and MN were Y_1_=0.2467x_1_+0.0317 [Standard deviation of intercept of regression line (Sa): 0.0352, Standard deviation of slope of regression line (Sb): 0.0118] and Y_2_=0.0677x_2_+0.0070 [Sa: 0.0100 and Sb: 0.0017] and 0.9998 and 0.9997, respectively, where x_1_ and x_2_ were the MSB and MN concentration (μg/mL) and Y_1_ and Y_2_ were the peak-area ratios of MSB to diazepam and MN to menatetrenone, respectively. Under the current assay conditions, LOD and LOQ for MSB were found to be 0.06 and 0.08 μg/mL. LOD and LOQ for MN were also found to be 0.04 and 0.06 μg/mL. 

The intra-day and inter-day precision and accuracy of the GC-FID method for both analytes were listed in Table 1. According to the analyzed results of MSB, the intra-day RSD % and RE were below 4.9 % and ± 2.4 % and inter-day RSD % and RE were below 7.5 % and ± 2.9 % in the QC concentration, respectively. According to the analysis results of MN, the intra-day RSD % and RE were below 5.9 % and ± 3.6 and inter-day RSD % and RE were below 7.4 % and ± 4.4 in the QC concentration, respectively. Precision and accuracy results of data obtained from both MSB and MN analyses results were similar and favorable.

**Table 1:** Precision and accuracy values of MSB and MN

**Method**	**Compound**	**Added ** **(g/mL)**	**Intra-day **			**Inter-day**		
			**FoundSD** **(g/mL)**	**Accuracy**	**Precision** **RSD %**	**FoundSD** **(g/mL)**	**Accuracy**	**Precision** **RSD %**
GC-FID	MSB	0.750	0.7320.036	-2.409	4.897	0.7470.056	-0.353	7.461
		5.000	4.8970.231	-2.056	4.713	4.8550.348	-2.891	7.162
		10.00	10.040.429	0.412	4.273	9.9450.571	-0.553	5.744
	MN	0.750	0.7230.042	-3.647	5.769	0.7830.040	4.391	5.126
		5.000	4.9270.292	-1.451	5.918	4.9930.369	-0.132	7.390
		10.00	10.080.278	0.760	2.761	9.9030.387	-0.997	3.915
HPLC-DAD	MSB	0.750	0.7520.018	0.220	2.376	0.7670.034	2.286	4.386
		5.000	4.9620.111	-0.768	2.228	5.0980.165	1.952	3.241
		10.00	10.170.077	1.715	0.754	10.360.232	3.578	2.235
	MN	0.750	0.7680.020	2.462	2.552	0.7450.058	-0.732	7.842
		5.000	5.2930.062	5.856	1.172	5.2350.071	4.709	1.353
		10.00	9.7510.455	-2.487	4.666	9.9400.579	-0.601	5.827

SD: standard deviation (n=6), RSD %: relative standard derivation % (n=6), Accuracy: (relative error).

Recovery in GC-FID method was determined by adding standard solution of MN and MSB to drug solution separately. To determine recovery of MSB, 0.75, 5 and 10 to 5 of MSB which were prepared from Libavit K^®^ ampoule and then the quantification of MSB was analyzed by GC-FID method. To determine recovery of MN, 0.75, 5 and 10 to 10 *i.e* 6.1 MN) which was prepared from Libavit K^®^ ampoule and then the quantification of MN was analyzed by this method. Experiments of each level were repeated six times. The results were given in Table 2 and the recovery values of MSB and MN were in the range of 94.1 %-97.9 % and 97.1 %-98.5 % with good accuracy, respectively.

**Table 2:** Recovery values of standard solution spiked in pharmaceutical preparation

**Method**	**Commercial preparation**	**Added ** **(g/mL)**	**FoundSD** **(g/mL)**	**Recovery (%)**	**RSD %**
GC-FID	Libavit K^®^ 5g/mL	0.750^b^	0.7210.040	96.24	5.544
		5.000^b^	4.7050.338	94.10	7.190
		10.00^b^	9.7920.485	97.92	4.956
	Libavit K^®^ 6.1^d^ g/mL	0.750^c^	0.7280.054	97.13	7.326
		5.000^c^	4.8780.226	97.56	4.638
		10.00^c^	9.8550.135	98.55	1.374
HPLC-DAD	Libavit K^®^ 5g/mL	0.750^b^	0.7360.038	98.20	5.217
		5.000^b^	4.7400.148	94.80	3.142
		10.00^b^	9.6950.431	96.95	4.452
	Libavit K^®^ 6.1^d^ g/mL	0.750^c^	0.7540.040	100.5	5.394
		5.000^c^	4.9480.345	98.96	6.975
		10.00^c^	9.8550.688	98.55	6.990

b :MSB standard solution.

c : MN standard solution.

d : MN equivalence concentration of 10 g/mL MSB. SD: standard deviation (n=6). RSD: relative standard derivation (n=6)

*Validation of HPLC-DAD method* *applied for analysis of MSB and MN*

**Figure 4:**
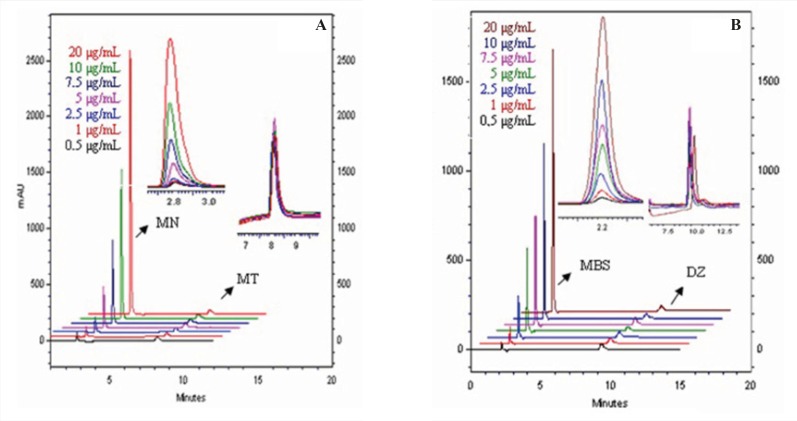
HPLC-DAD chromatograms of obtained concentration in calibration graph of (A) MN standard solution of containing menatetrenone (B) MSB standard solution of containing diazepam

The developed GC-FID method appears to be specific for MN and MSB in drug products. HPLC methods were faster than GC-FID method in terms of their elution time of analytes. We have studied on two HPLC analyses with different mobile phase and wavelength. In the first one, we analyzed MSB and diazepam. The retention times of MSB and diazepam were 2.2 min and 9.3 min, respectively. The second analysis was applied to determine MN and menatetrenone. The retention times of MN and menatetrenone are 2.7 min and 8.05 min, respectively. While, the total run time in first analysis was 9.5 min, this time in the second analysis was 8.1 min. The overlay of typical chromatogram obtained from the HPLC analysis of MSB and MN in the concentration of 0.5-20 μg/mL containing its own IS was shown in Figure 4A and 4B. The calibration curves were linear in the same concentration range (0.5–20 μg/mL) with GC-FID for both analytes. The regression equations for MSB and MN were Y_1_=0.6348x_1_+0.0502 [Sa: 0.0789 and Sb: 0.0186] and Y_2_=1.0498x_2_+0.0656 [Sa: 0.1542 and Sb: 0.0659] (x_1_: MSB concentration, x_2_: MN concentration, Y_1_: the peak-area ratios of MSB to diazepam Y_2_: the peak-area ratios of MN to menatetrenone) with a correlation coefficients (*R*) of 0.9999 and 0.9992, respectively. 

LOD value of MSB was found to be 0.005 μg/mL, this parameter was determined to be 0.01 μg/mL for MN. LOQ value of both MSB and MN was also 0.015 μg/mL.

The results of intra-day and inter-day precision and accuracy obtained with HPLC-DAD for both analytes method were shown in Table 1. According to the analyzed results of MSB, the intra-day RSD % and RE were below 2.4 % and ± 1.7 and inter-day RSD % and RE were below 4.4 % and ±3.6 in the QC concentration, respectively. According to the analysis results of MN, the intra-day RSD % and RE were below 4.6 % and ± 5.8 and inter-day RSD and RE were below 7.8 % and ± 4.7 in the QC concentration, respectively.

Recovery in HPLC-DAD method was also determined by adding the standard MN and MSB separately. To determine recovery of MSB, 0.75, 5 and 10 to 5 of MSB which was prepared from Libavit K^®^ ampoule and then the quantification of MSB was analyzed by GC-FID method. To determine recovery of MN, 0.75, 5 and 10 to 10 *i.e *6.1 MN) of prepared from Libavit K^®^ ampoule and then the quantification of MN was analyzed by that way. Experiment of each level was repeated six times. The results were given in Table 2 and the recovery values of MSB and MN were in the range of 94.8 %-98.2 % and 98.5 %-100.5 % with good accuracy, respectively.

The related stability experiment indicated that MSB was stable for 24 h at 4 °C and 25 °C and at least 60 h at -20 °C while MN was stable for 24 h at 25 °C and at least 60h at 4 °C and -20 °C. The stability results were summarized in Table 3.

**Table 3:** Stability values of MN and MSB in standard solution

**Stability ** **(Recovery % SD)**	**Compound**	**Added** **(g/mL)**	**6 h**	**24 h**	**48 h**	**60 h**	
Room temperature stability +25°C	MSB	0.750	95.35±0.792	97.50±2.703	(-)	(-)	
		5.000	98.15±3.432	101.2±2.381	(-)	(-)	
		10.00	92.67±1.138	94.58±3.391	(-)	(-)	
	MN	0.750	97.46±1.611	96.35±1.409	(-)	(-)	
		5.000	99.50±4.511	98.90±1.729	(-)	(-)	
		10.00	99.62±3.312	106.9±2.238	(-)	(-)	
Refrigeratory stability, +4°C	MSB		24 h	48 h	60 h	
		0.750	95.59±4.183	88.90±1.549	81.34±3.287	
		5.000	97.49±3.899	83.03±3.858	79.62±1.764	
		10.00	97.03±0.715	87.12±3.098	81.50±1.907	
	MN	0.750	102.3±3.125	104.5±4.322	113.3±0.930	
		5.000	101.9±3.032	102.4±1.032	94.49±2.990	
		10.00	101.8±1.628	104.3±3.814	97.40±2.060	
Frozen stability - 20°C	MSB		24 h	48 h	60 h	
		0.750	97.52±3.531	95.60±2.432	94.04±1.185	
		5.000	99.26±3.286	96.17±3.400	95.66±1.588	
		10.00	95.61±2.730	95.70±2.894	95.09±2.974	
	MN	0.750	98.67±0.971	106.9±1.486	101.7±1.456	
		5.000	100.9±3.325	108.2±3.974	100.3±4.414	
		10.00	99.57±3.672	99.85±0.413	97.47±0.349	

SD: Standard deviation (n=3). (-): any peaks were not observed.

*Ratio of converting of MSB to MN*

To determine the conversion rate of MSB to MN with HPLC-DAD and GC-FID studies, MSB and MN were added into 0.01 M HCl solution separately. In order to provide the same concentration ranges (0.061-12.2 µg/mL for HPLC-DAD method and 0.61-36.6 µg/mL for GC-FID method) of each of the two analytes, the MSB was converted to MN as described above section and then extracted with *n*-hexane. 

*For GC-FID method: *The recoveries of MN and IS added to 0.01 M HCl were 98.9 % (RSD %: 2.7% n=6) and 96.8 % (RSD %: 2.1 %, n=6), respectively. The recoveries of MSB converted to MN and IS were 96.9 % (RSD %: 6.3 %, n=6) and 98.1 % (RSD%: 4.1%, n=6), respectively. Consequently, the conversion rate of MSB to MN was determined to be 98.1% (RSD %: 6.0 %, n=6).

*For HPLC method: *The recoveries of MN and IS added to 0.01 M HCl were 101.1 % (RSD %: 1.7 %, n=6) and 96.1 % (RSD %: 2.5 %, n=6), respectively. The recoveries of MSB converted to MN and IS were 98.1 % (RSD %: 5.1 %, n=6) and 95.9 % (RSD %: 1.8 %, n=6), respectively. The conversion rate of MSB to MN was determined to be 97.2 % (RSD %: 6.2 %, n=6).

*Application and comparison of the p* *roposed methods*

The developed GC-FID (method 1) and HPLC-DAD (method 2) methods were applied to determine the MSB in the Libavit K^®^ (from Mefar Drug Company, Turkey) ampoule. Quantitative analysis of MSB in commercial pharmaceutical was performed in both direct analysis of MSB (analysis 1) and analysis of MN by converting MSB to MN (analysis 2). 

**Figure 5:**
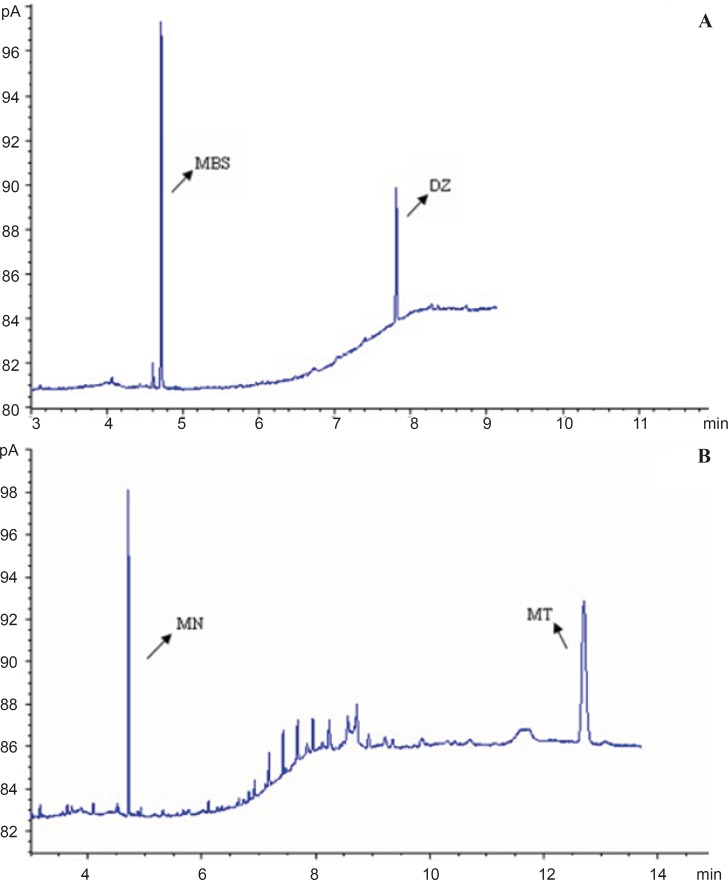
GC-FID Chromatogram of solutions of Libavit K^®^ ampoule containing MSB (A) direct analysis of MSB (B) analysis of MN by converting MSB to MN

**Figure 6:**
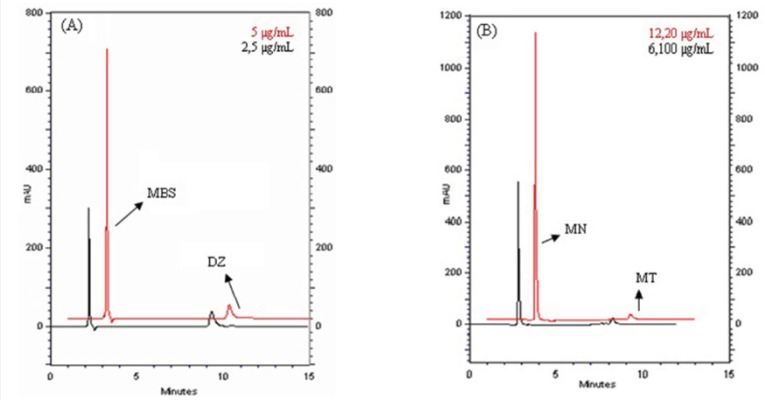
HPLC Chromatogram of solutions of Libavit K^®^ ampoule containing MSB (A) direct analysis of MSB (B) analysis of MN by converting MSB to MN

GC-FID chromatograms obtained from direct analysis of MSB and analysis of MN by converting MSB to MN in drug solution were shown in Figure 5A and 5B, respectively. HPLC-DAD chromatograms obtained from direct analysis of MSB and analysis of MN by converting MSB to MN in drug solution were shown in Figure 6A and 6B, respectively.

Intra-method comparisons were performed by student-t test (p-value is <0.05). According to data obtained in this analysis, there was no significant difference between method 1 and 2 (p= 0.622 for direct analysis and p= 0.267 for conversion analysis). For inter-group comparison,* student-t *test was also used for two independent analysis methods. According to data obtained in this analysis, there is no significant difference between analysis 1 and 2 (p= 0.622 for GC-FID method and p= 0.267 for HPLC-DAD method). Furthermore, the data in Table 4 indicated that the MSB (or MN) contents measured by the proposed methods were in good agreement with the values supplied by the manufacturers.

**Table 4:** Analysis of Libavit K ampule (20 mg/mL).

**Method**		**n**	**Found** e **SD** ** (mg)**	**Recorvery** **(%)**	**R.S.D** ^a^ **(%)**	**Confidence Interval**	**t** ***-*** **values**	**t** ***-*** **values**
GC-FID	direct analysis of MSB	12	19.660.955	98.31	4.855	93.29-109.6	* t*_c_=0.435 (P=0.549)	* t*_c_=-1.450 (P=0.622. for direct analysis) *t*_c_=-0.038 (P=0.267.for conversion analysis)
	analysis of MN by converting MSB to MN	12	19.871.414	99.38	7.115	83.20-113.0		
HPLC-DAD	direct analysis of MSB	12	19.090.970	95.46	5.081	87-2-102.5	* t*_c_=2.191 (P=0.161)	
	analysis of MN by converting MSB to MN	12	19.850.726	99.29	3.656	92.44-105.2		

n: number of determination. SD: standart deviation. RSD: Relative standard derivation . t_c_: calculated t values. H_o_: Hypothesis: no statitically significant difference exists between two methods t_t_ t_c; _H_o _hypothesis in accepted (=0.05)

e : MSB concentration.

## Conclusions

Generally, researchers have used C_18_ column for analysis of vitamin K3 with HPLC method. For example, MSB was analyzed with C_18_ column by Ruiz *et al.* (18) and Liu et al (19). We achieved to measure both MN and MSB by using C_8_ column which is differentiated from the literature. 

There was only one article related with analysis of vitamin K3 with GC-FID. Nevertheless, we could not obtain detailed information from that article. Only we know that MSB is directly analyzed. Because of that reason, our study is the first method which analyzes converted form of MSB (MN) by GC-FID method. 

In the literatures mentioned above, linearity range was determined as 0.1-10 μg/mL (18) and 0.035-15 μg/mL (19). Linearity range of our chromatographic methods (GC-FID and HPLC) was extended with these studies (0.5-20 μg/mL). LOD values in literature were found 0.8 ng/mL (18), 15 ng (19) and 2.5 μg/mL (17). HPLC-DAD method developed by us was as sensitive as the above- mentioned methods (Table 1). Although LOD concentration of proposed GC-FID method was not as sensitive as our HPLC-DAD and other methods in literature, it has been successfully and equally applied for determination of MN and MSB at pharmaceutical preparation. Finally, we suggest simple, accurate, precise GC-FID and HPLC-DAD methods that requires no extra equipment and also these methods can be directly and easily applied for both direct analysis of MSB and analysis of MN by converting MSB to MN in commercial pharmaceutical containing MSB.
